# Ductal Ngn3-expressing progenitors contribute to adult β cell neogenesis in the pancreas

**DOI:** 10.1016/j.stem.2023.02.005

**Published:** 2023-04-06

**Authors:** Christopher Gribben, Christopher Lambert, Hendrik A. Messal, Ella-Louise Hubber, Chloe Rackham, Ian Evans, Harry Heimberg, Peter Jones, Rocio Sancho, Axel Behrens

(Cell Stem Cell *28*, 2000–2008.e1–e4; November 4, 2021)

In the originally published version of our manuscript, in Figure 3, we made an error when labeling the axes in Figures 3C and 3G. In Figure 3C, four sections were analyzed per mouse, and the numbers given are the *average* numbers of positive cells *per section*, with each data point representing one animal and *not* positive cells per mouse. In Figure 3G (third column), only Ngn3-CreERT; AKITA mice are analyzed, without any lineage tracing, with cells expressing INS or SST quantified. To address this, we have now updated the graph labels, and an updated Figure 3 is included below. We apologize for the oversight and for any confusion caused.

**Figure 3:**
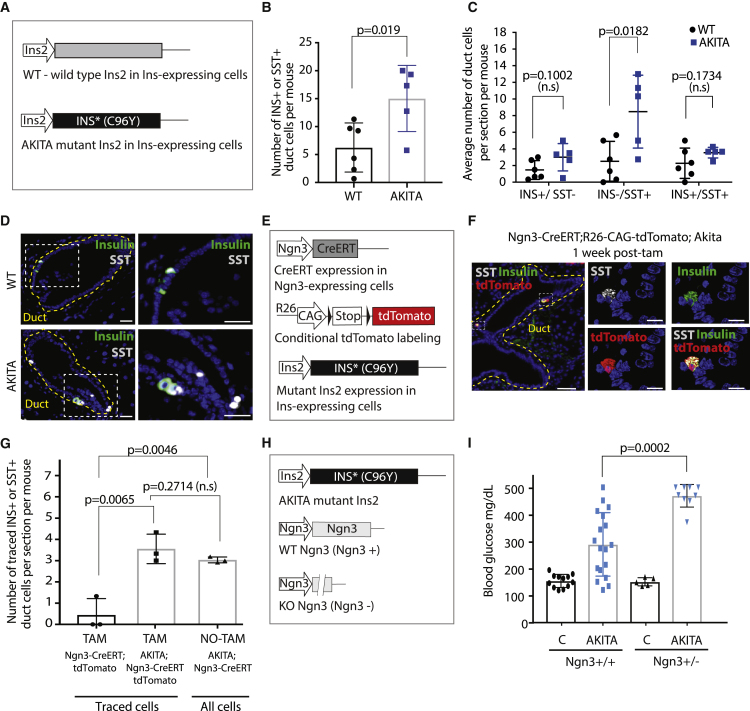
Ngn3+ ductal cell contribution to the endocrine cell population is increased during diabetes (corrected).

